# Novel molecular components involved in callose-mediated Arabidopsis defense against *Salmonella enterica* and *Escherichia coli* O157:H7

**DOI:** 10.1186/s12870-019-2232-x

**Published:** 2020-01-08

**Authors:** Paula Rodrigues Oblessuc, Cleverson Carlos Matiolli, Maeli Melotto

**Affiliations:** Department of Plant Sciences, University of California, One Shields Avenue, Davis, CA 95616 USA

**Keywords:** *Salmonella enterica*, Shiga toxin-producing *Escherichia coli*, *Pseudomonas syringae*, Arabidopsis mutants, Callose deposition, MAMP-triggered immunity

## Abstract

**Background:**

Food contamination with *Salmonella enterica* and enterohemorrhagic *Escherichia coli* is among the leading causes of foodborne illnesses worldwide and crop plants are associated with > 50% of the disease outbreaks. However, the mechanisms underlying the interaction of these human pathogens with plants remain elusive. In this study, we have explored plant resistance mechanisms against these enterobacteria and the plant pathogen *Pseudomonas syringae* pv. *tomato* (*Pst*) DC3118, as an opportunity to improve food safety.

**Results:**

We found that *S. enterica* serovar Typhimurium (STm) transcriptionally modulates stress responses in Arabidopsis leaves, including induction of two hallmark processes of plant defense: ROS burst and cell wall modifications. Analyses of plants with a mutation in the potentially STm-induced gene *EXO70H4* revealed that its encoded protein is required for stomatal defense against STm and *E. coli* O157:H7, but not against *Pst* DC3118. In the apoplast however, EXO70H4 is required for defense against STm and *Pst* DC3118, but not against *E. coli* O157:H7. Moreover, EXO70H4 is required for callose deposition, but had no function in ROS burst, triggered by all three bacteria. The salicylic acid (SA) signaling and biosynthesis proteins NPR1 and ICS1, respectively, were involved in stomatal and apoplastic defense, as well as callose deposition, against human and plant pathogens.

**Conclusions:**

The results show that EXO70H4 is involved in stomatal and apoplastic defenses in Arabidopsis and suggest that EXO70H4-mediated defense play a distinct role in guard cells and leaf mesophyll cells in a bacteria-dependent manner. Nonetheless, EXO70H4 contributes to callose deposition in response to both human and plant pathogens. NPR1 and ICS1, two proteins involved in the SA signaling pathway, are important to inhibit leaf internalization and apoplastic persistence of enterobacteria and proliferation of phytopathogens. These findings highlight the existence of unique and shared plant genetic components to fight off diverse bacterial pathogens providing specific targets for the prevention of foodborne diseases.

## Background

Foodborne diseases caused by human pathogens have profound social and economic impacts. Despite several safety measures taken to prevent disease outbreaks, it is estimated that 600 million (1 in 10) people are affected worldwide every year [1]. According to the US Foodborne Disease Outbreak Surveillance System (FDOSS), there were 972 outbreaks associated with raw produce during 1998–2013 in the US, resulting in 34,674 illnesses events, 2315 hospitalizations, and 72 deaths [2]. The most common etiologic agents identified in these foodborne disease outbreaks were norovirus (54%), *Salmonella enterica* (21%) and Shiga toxin-producing *Escherichia coli* (10%) [2]. Therewith, the US Food Safety Modernization Act (FSMA) was established in 2011, seeking to change the food safety system by shifting the focus from foodborne illness response to disease prevention. Thus, it is imperative to understand the biological processes involved in the human bacterial pathogen-plant interactions to prevent, or at least minimize, enteropathogen colonization of fresh produce.

It has been previously thought that plants could be passive vectors for human pathogens. However, recent studies support the hypothesis that *S. enterica* and enterohemorrhagic *E. coli* are capable of colonizing plant tissues and plants can activate immune responses upon bacterial colonization [3–23]. These bacteria are able to survive in the leaf apoplast of a wide range of plant species, including cilantro (*Coriandrum sativum*), lettuce (*Lactuca sativa* L.), *Arabidopsis thaliana*, tomato (*Solanum lycopersicum*), spinach (*Spinacia oleracea*), *Nicotiana benthamiana*, and basil (*Ocimum basilicum*) [6, 11, 22, 24–29]. Once inside the leaf apoplast, these bacteria remain protected from common sanitation treatments of edible leaves [30, 31], posing a risk to reach their human host.

Endophytic survival of bacterial pathogens of humans seems to be regulated by the plant immune system reviewed by [32–34]. Microbe-associated molecular patterns (MAMPs), such as the motor protein flagellin in bacteria [35], can induce plant basal defenses known as MAMP-Triggered Immunity (MTI) [36]. For instance, Seflg22, a 22 amino acid-peptide derived from the *S. enterica* serovar Typhimurium (STm) flagellin, is perceived by Arabidopsis, *Nicotiana benthamiana*, tomato, and *Medicago truncatula* [23, 37, 38], and the *E. coli* flagellin-derived peptide flg15^*. coli*^ is biologically active in tomato, spinach, and Arabidopsis plants [15, 18, 39–41]. Moreover, STm and *E. coli* O157:H7 induce stomatal closure, a well-known MTI response, in several plant species [17, 22, 42] and lead to MTI-associated transcriptional changes in plants [15, 32, 38, 43–45]. In fact, a large set of Arabidopsis genes is regulated by *E. coli* O157:H7 in a flagellin-dependent manner, evidenced by comparative transcriptome analysis between the wild type and a *fls2* mutant (deficient in flagellin perception) inoculated with *E. coli* O157:H7 or the *E. coli* flagellin deficient mutant TUV86–2 *fliC* [43, 45]. Overall, these studies indicate that both STm and *E. coli* induce MTI in plants, although the mechanisms downstream of enterobacterium perception are still elusive.

In this study, we explored the plant transcriptional modulation mediated by STm and used Arabidopsis genetic resources to again insights on the molecular mechanisms activated during plant colonization with STm and *E. coli* O157:H7. Additionally, we sought to establish correlations with the model system Arabidopsis-*Pseudomonas syringae* pv*. tomato* (*Pst*). We demonstrate that EXO70H4 (EXOCYST SUBUNIT EXO70 FAMILY PROTEIN H4) participates in the stomatal defense in response to both enterobacteria, while in the leaf apoplast it is involved in the immune response only against STm and *Pst.* Therefore, EXO70H4 seems to function in a bacterium-specific manner, depending on the plant cell type. Moreover, we show that plant basal defense against STm 14,028 s, *E. coli* O157:H7, and *Pst* DC3118 require the salicylic acid (SA) biosynthesis and signaling proteins ICS1 (ISOCHORISMATE SYNTHASE 1) and NPR1 (NON-EXPRESSER OF PATHOGENESIS-RELATED GENE 1), respectively. Interestingly, EXO70H4, ICS1, and NPR1 contribute to leaf callose deposition in response to all of these bacteria. Our results indicate that EXO70H4 is a novel component of plant defense against pathogens. Additionally, we demonstrate that SA-mediated immunity is a common plant defense response to both entero- and phyto-bacteria that involves the induction of callose deposition through EXO70H4. These findings represent an advancement of our current understanding of the plant genetic mechanisms that ultimately protect leaves against successful bacterial colonization.

## Results

### Global gene expression analysis reveals plant processes modulated by STm in Arabidopsis

To identify specific genetic mechanism of Arabidopsis defenses towards STm persistence, we performed a transcriptomic analysis of leaves inoculated with the STm strain SL1344 using the Affymetrix GeneChip ATH1 (Thermo Scientific, Rockford, IL). Z-ratio normalization of array intensity data [46] showed a normal distribution of relative gene expression (STm- versus mock-treated samples) and revealed the significant differentially expressed genes within the 2% extremes of the bell curve (Additional file 1). Linear regression between gene expression calculated as Z-ratio and Log_2_ fold changes showed a high correlation (R^2^ = 0.9676) (Additional file 1), where both extreme Z-ratio values coincided with both extreme Log_2_ fold change values. Using these two methods to calculate relative gene expression, we identified 585 differentially expressed genes, in which 310 were up-regulated and 275 were down-regulated upon STm SL1344 inoculation (Additional file 2; ID NASCARRAYS-674). To increase the confidence for the identification of differentially expressed genes, we validated the microarray analysis using Reverse Transcriptase-quantitative PCR (RT-qPCR). The relative expression level of nine randomly selected genes from the microarray dataset (four up-regulated, three non-regulated, and two down-regulated; Additional file 2) showed the same expression patterns calculated by RT-qPCR (Additional file 3), indicating that the microarray analysis is robust enough for calling differentially expressed genes.

**Fig. 1:**
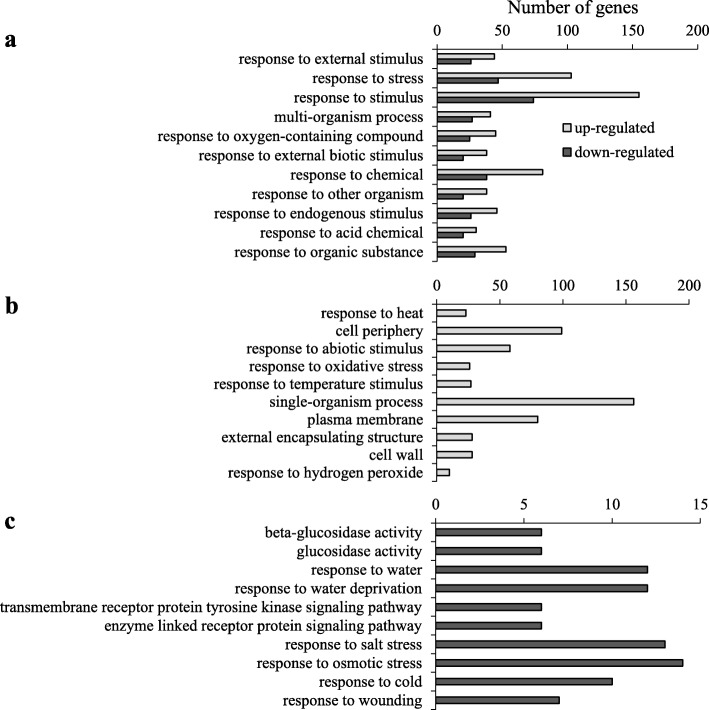
Gene Ontology (GO) enrichment analysis reveled processes modulated by STm SL1344 in Arabidopsis leaves. **a** All GO terms identified as significantly abundant in both induced and repressed gene datasets. **b** and **c** The top ten most enriched GO terms found in either induced (**b**) or repressed (**c**) gene dataset. Statistical significance for the GO enrichment analysis was detected with the Fisher’s exact test followed by the Yekutieli-False Discovery Rate multiple test correction (FDR < 0.05). Additional file 4 contains the complete list of significantly enriched GOs

Gene Ontology (GO) single enrichment analysis (SEA) of genes differentially regulated by STm SL1344 allowed the identification of metabolic processes modulated in bacterium-inoculated samples. We identified 144 and 25 GO terms significantly more abundant (FDR *p* < 0.05) in the up-regulated and down-regulated gene sets, respectively, as compared to the Arabidopsis reference gene model available at TAIR10 (Arabidopsis.org) (Additional file 4). Eleven GO terms were significantly enriched in both up- and down-regulated gene sets (Fig. 1a). Among them, two are parental terms, ‘multi-organism process’ and ‘response to stimulus’, and the other nine are child terms of the latter (Additional file 5). This suggest that STm SL1344 modulated plant stress responses.

GO terms exclusively associated with up-regulated genes support the hypothesis of activation of defense responses by STm SL1344 in Arabidopsis leaves (Additional file 4). Among them, the GO terms ‘response to oxidative stress’ and ‘response to hydrogen peroxide’ indicate that STm SL1344 can induce reactive oxygen species (ROS) burst, a well-known immune response in plants. In addition, the GO terms ‘cell periphery’, ‘plasma membrane’, ‘external encapsulating structure’ and ‘cell wall’ were enriched categories exclusively present in the STm SL1344-induced genes dataset, suggesting that this bacterium triggers cell wall modifications in Arabidopsis. Interestingly, ‘response to heat’ and ‘response to temperature stimulus’ were also among the up-regulated enriched GO terms, which include several heat shock proteins (Fig. 1b; Additional file 4). These proteins function as molecular chaperones and play an important role in the quality control of protein receptors at the plasma membrane during plant-microbe interaction [47].

We also identified GO terms that are exclusively overrepresented in the STm SL1344 down-regulated gene dataset (Additional file 4). Among them, the ‘beta-glucosidase activity’ and ‘glucosidase activity’ categories (Fig. 1c) indicate that STm SL1344 down-regulates the hydrolysis of glucosyl compounds. Moreover, the GO terms ‘response to water’, ‘response to water deprivation’, ‘response to salt stress’, and ‘response to osmotic stress’ suggest that STm SL1344 repress drought and salt stress responses in Arabidopsis. The GO term ‘response to cold’ (Fig. 1c) covers genes mainly related to glycosyl hydrolysis and drought/salt stress responses (Additional file 4), reinforcing the down-regulation of these plant responses by STm SL1344. Furthermore, two GO terms related to signal transduction, ‘transmembrane receptor protein tyrosine kinase signaling pathway’ and ‘enzyme linked receptor protein signaling pathway’, were enriched within the down-regulated genes, covering six leucine-rich repeat protein kinases related to plant development.

Overall, we can conclude that STm SL1344 causes transcriptional modulations in Arabidopsis leaves, affecting plant innate immune responses and cell growth, mainly by the induction of ROS burst and cell wall modifications, as well as by repressing glucosyl compounds hydrolysis, drought and salt stress responses, and signaling responses mediated by developmental-related receptors.

### EXO70H4 is a novel protein that contribute to both stomatal- and apoplast-based defense in a bacterium-specific manner

**Fig. 2:**
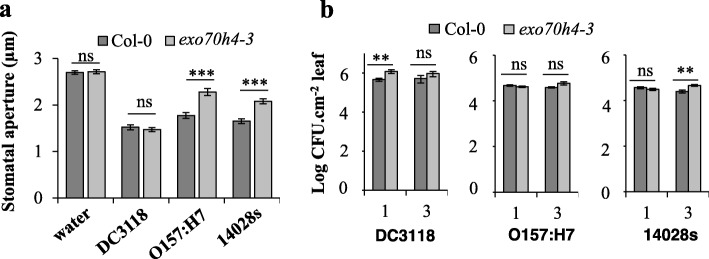
EXO70H4 contribute to both stomatal- and apoplast-based defense in a bacterium-specific manner. **a** Stomatal aperture width in Arabidopsis leaves was measured at 2 h post inoculation with *Pst* DC3118, *E. coli* O157:H7, or STm 14,028 s at a concentration of 1 × 10^8^ CFU.mL^− 1^. Results are shown as mean of two independent biological replicates (*n* = 120 ± SE). **b** Bacterial population was evaluated at one and three days after vacuum-inoculation with *Pst* DC3118, *E. coli* O157:H7, or STm 14,028 s at a concentration of 1 × 10^6^ CFU.mL^− 1^. Results are shown as the average of two independent biological replicates (n = 12 ± SE). Note that human bacterial growth within plants is very consistent as reflected by the small error bars. Statistical difference between means (mutant versus Col-0) was detected with two-tailed Student’s t-test (** = *p* < 0.01; *** = *p* < 0.001; ns = non-significant). CFU = colony forming unit

Transcriptome analysis revealed that STm SL1344 induces the *EXO70H4* gene (Additional file 2). Therefore, we evaluated the possible role of EXO70H4 in the Arabidopsis interaction with STm, as well as with *E. coli* O157:H7 and *Pst* DC3118. For these experiments, we used the *exo70h4–3* mutant that has a T-DNA insertion in the coding sequence of the *EXO70H4* gene leading to the synthesis of a truncated mRNA (Additional file 6) [48];. This mutant plant had a defective stomatal closure only after inoculation with the human pathogens STm 14,028 s and *E. coli* O157:H7 (Fig. 2a). All three bacterial induced a significant reduction in the in the average stomatal aperture width in both Col-0 and *exo70h4–3* plants (Additional file 7). We also evaluated the bacterial population dynamics in leaves of the *exo70h4–3* mutant and observed that *Pst* DC3118 population was higher than that of Col-0 only in the first day after inoculation (DAI), whereas the STm 14,028 s population persisted at a high level at the third DAI (Fig. 2b). The EXO70H4-mediated apoplastic defense seems to be bacterium specific, as *E. coli* O157:H7 showed same level of survival inside *exo70h4–3* leaves in comparison to Col-0 (Fig. 2b). Therewith, EXO70H4 may have a function in the guard cell response specifically to enterobacteria, whereas in the leaf mesophyll, EXO70H4 activity may be important for defense against *Pst* DC3118 and STm 14,028 s, but not against *E. coli* O157:H7.

### EXO70H4 contributes to bacterium-induced callose deposits, but not ROS burst

**Fig. 3:**
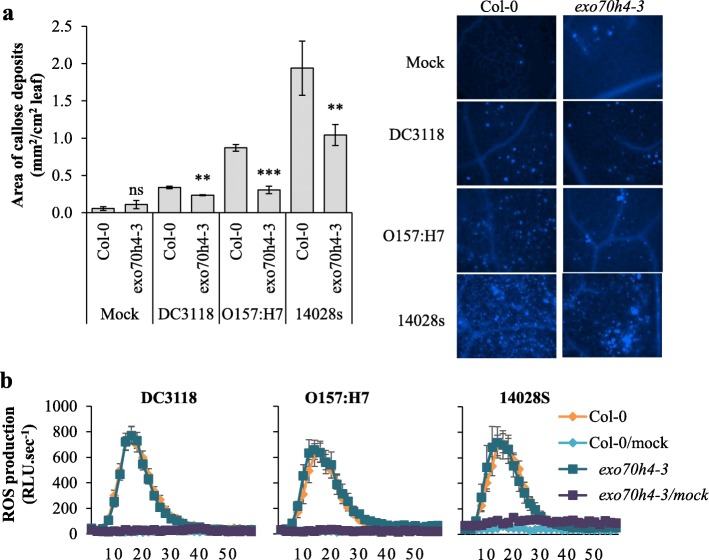
EXO70H4 contributes to bacterium-induced callose deposits, but not ROS burst. **a** Callose deposits area was measured in each genotype after four- to five-week-old plants were syringe-infiltrated water as a mock control or with 1 × 10^8^ CFU.mL^− 1^ of *Pst* DC3118, STm 14,028 s, or *E. coli* O157:H7. Results are shown as average of three to four biological replicate (*n* = 18 to 37 ± SE) and the experiment was repeated twice with similar results. Statistical difference between the means (wild type versus mutant plant) was calculated using the two-tailed Student’s *t*-test (** = *p* < 0.01; *** = *p* < 0.001; ns = non-significant). The pictures on the left are representative of each plant genotype per treatment. **b** Leaf discs of four- to five-week-old plants were incubated with control solution or 1 × 10^8^ CFU.mL^− 1^ of boiled *Pst* DC3118, STm 14,028 s, or *E. coli* O157:H7. Results are shown as the average of luminescence per genotype in three independent biological replicates (*n* = 21 to 37 ± SE). Luminescence was recorded over 60 min using a Synergy™ H1 microplate reader and the Biotek Gen5 (Biotek) software. RLU = Relative Light Units

We have found that STm SL1344 transcriptionally modulates ‘cell wall’ processes in Arabidopsis leaf (Fig. 1b) and EXO70H4 was shown previously to participate on the callose deposition on leaf trichomes [48]. As callose deposition is a hallmark plant basal immune response to pathogens reviewed by [49], we assessed the extent of callose production in the *exo70h4–3* mutant after bacterial inoculation. Reduced callose deposition was observed in *exo70h4–3* as compared to Col-0 after inoculation with any of the three bacteria (Fig. 3a), suggesting that EXO70H4 contributes to the formation of callose in leaves under biotic stress.

ROS burst is another hallmark of plant basal defense response [50], and our transcriptome analysis revealed that STm modulates plant ‘response to oxygen-containing compounds’, inducing ‘response to oxidative stress’ and ‘response to hydrogen peroxide’ (Fig. 1). Therefore, we measured bacterium-induced ROS production in leaves of Col-0 and *exo70h4–3* mutant plants to verify whether ROS could contribute to the enhanced susceptibility observed in *exo70h4–3* plants (Fig. 2b). Mock-treated leaves did not show a ROS burst (Fig. 3b), whereas all three bacteria induced similar level of ROS in both Col-0 and *exo70h4–3* (Fig. 3b). These findings indicate that, although both enterics and the phytopathogen activate this plant defense, EXO70H4 has no role in this process.

### Defense against human pathogens, similar to plant pathogens, requires the SA signaling components ICS1 and NPR1

**Fig. 4:**
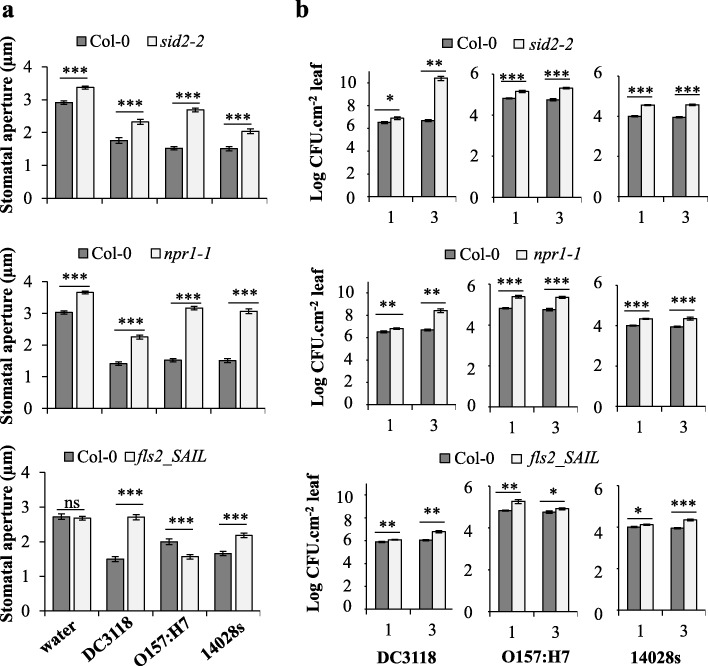
A functional SA signaling pathway is required to promote bacterial-triggered stomatal closure and apoplastic immunity. Arabidopsis mutants *sid2–2*, *npr1–1* and *fls2_SAIL* have impaired stomatal and apoplastic defenses against *Pst* DC3118, *E. coli* O15:H17, and STm 14,028 s. **a** Stomatal aperture width was measured 2 h post inoculation with each bacterium at a concentration 1 × 10^8^ CFU.mL^− 1^. Results are shown as the average of two independent biological replicates (n = 120 ± SE). **b** Bacterial population sizes evaluated one and three days after inoculation. Plants were vacuum-inoculated with each bacterium at a concentration of 1 × 10^6^ CFU.mL^− 1^. Results are shown as the average of two independent biological replicates (n = 12 ± SE). Note that very small error bars are observed for bacterial growth of mainly human pathogen due to the high reproducibility of the assay. Statistical significance between means (mutant versus Col-0) was detected with two-tailed Student’s *t*-test (* = *p* < 0.05; ** = *p* < 0.01; *** = *p* < 0.001; ns = non-significant). CFU = colony forming unit

Plant defense to bacteria, including callose biosynthesis, is known to require a functional SA pathway reviewed by [49]. Thus, we evaluated plants with impaired SA biosynthesis through a mutation in the *ICS1* gene, *sid2–2* [51], and SA signaling through a mutation in the *NPR1* gene, *npr1–1* [52]. Homozygosity in the *sid2–2* and *npr1–1* plant lines were confirmed by PCR (Additional file 6). *Pst* DC3118, *E. coli* O157:H7, and STm 14,028 s did not induce stomatal closure in *sid2–2* and *npr1–1* mutants to the same extent as they did in Col-0 plants (Fig. 4a); although all three bacteria induced a significant stomatal closure in Col-0, *sid2–2*, and *npr1–1* as compared to the water control (Additional file 7), indicating that stomatal immunity is not completely abolish in these mutants. Interestingly, the water-treated mutant leaves had a significantly wider stomatal aperture width when compared with stomata of Col-0 leaves (Fig. 4a), indicating that SA synthesis and signaling also control stomatal aperture in the absence of biotic stress (i.e., bacterium inoculation). All three bacteria maintained a larger apoplastic population in the *npr1–1* and *sid2–2* mutants than in Col-0, where the population of the phytopathogen *Pst* DC3118 increased and the population of *E. coli* O157:H7 and STm 14,028 s persisted at a higher level in these mutant than in the wild type plants throughout the experimentation time (Fig. 4b). These results indicate that SA biosynthesis and signaling through proteins ICS1 and NPR1, respectively, are not only contributes to Arabidopsis defenses against *Pst* as previously described [42, 51, 53], but also to leaf stomatal and apoplastic defenses against the bacterial human pathogens *E. coli* O157:H7 and STm 14,028 s.

SA-mediated immunity can be triggered upon detection of MAMPs by transmembrane receptors in the guard cells, such as the FLS2 (FLAGELLIN SENSING 2) that perceive flagellin from bacteria [36, 42, 54, 55]. Therefore, we tested whether flagellin perception through FLS2 plays a role in Arabidopsis defense against STm 14,028 s and *E. coli* O157:H7 as well. As previously shown [42, 55], the *fls2_SAIL* mutant (Additional file 6) [56]; has impaired stomatal closure in response to *Pst* DC3118 (Fig. 4a). Interestingly, *E. coli* O157:H7 triggered enhanced stomatal immunity in *fls2_SAIL*, while STm 14,028 s failed to induce stomatal closure in *fls2_SAIL* mutant to the same extent as in Col-0 (Fig. 4a). These findings indicate that FLS2 is sufficient for Arabidopsis to recognize *Pst* DC3118 and STm 14,028 s and activate stomatal closure; however, other MAMPs may redundantly contribute to *E. coli* O157:H7-triggered stomatal closure. Interestingly, *Pst* DC3118, but not STm 14,028 s and *E. coli* O157:H7, failed to induce stomatal closure in *fls2-SAIL* (Additional file 7). Furthermore, apoplastic defense in the *fls2_SAIL* mutant was compromised in response to all three bacteria, as higher bacterial population was observed inside of this mutant leaf when compared to Col-0 (Fig. 4b). This finding indicates that the FLS2 receptor has a significant role in the perception of *Pst*, STm 14,028 s, and *E. coli* O157:H7 by the mesophyll cells, at least in the first three days of interaction.

Altogether, these results suggest that SA signaling components ICS1 and NPR1, as well as the MAMP receptor FLS2, function in plant defense against human pathogens in a similar manner to that of against phytopathogen *Pst*.

### ICS1 and NPR1 contribute to enterobacterium-induced callose production

**Fig. 5:**
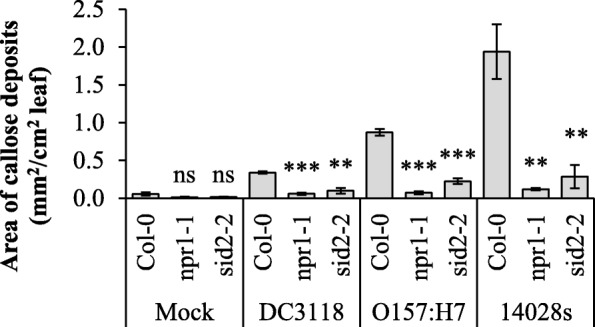
SA signaling are involved in enterobacterium-induced callose production. Callose deposits area was measured in leaves of four- to five-week-old plants after syringe-infiltration with water as a mock control or 1 × 10^8^ CFU.mL^− 1^ of *Pst* DC3118, STm 14,028 s, or *E. coli* O157:H7. Results are shown as mean (n = 18 to 37 ± SE). Statistical difference between the means (Col-0 versus mutant plant) was detected using two-tailed Student’s *t*-test (** = *p* < 0.01; *** = *p* < 0.001; ns = non-significant)

Bacterial infection is known to induce cell wall thickening through callose deposition in Arabidopsis [57], a process that is associated with SA signaling [58]. We verified that *Pst* DC3118, *E. coli* O157:H7, and STm 14,028 s induce callose deposition in the wild type plant Col-0 (Fig. 3a). Additionally, *sid2–2* and *npr1–1* mutants showed enhanced susceptibility to STm 14,028 s and *E. coli* O157:H7 (Fig. 4b). Thus, we tested whether ICS1 and NPR1 are also relevant to callose deposition triggered by these human pathogens by evaluating the area of callose deposits produced in the respective mutant leaves. A significant decreased in callose deposition was observed in both mutants, *sid2–2* and *npr1–1*, in response to all bacteria (Fig. 5) suggesting that SA biosynthesis via ICS1 and signaling by NPR1 via callose production is a shared mechanism of Arabidopsis defense against enterobacteria and the phytopathogen *Pst*.

## Discussion

It is well-known that plants can defend themselves against a wide range of microbes through the activation of innate immune responses. Plant defense against plant pathogens has been reported extensively and robust markers for this defense are widely accepted to track these responses. For instance, perception of MAMPs and DAMPs by host transmembrane receptors, ROS production, callose deposition, and hormonal crosstalk are few of widely studied and important defense mechanisms used by plants reviewed by [59]. Although plants are able to respond to human pathogenic bacterial colonization, the molecular mechanisms underlying plant interaction with enterobacteria is a major unexplored area of research. Our study contributes to this field by assessing novel molecular components of plant resistance and determining whether these molecules are associated with hallmark plant defense mechanisms thereby reducing persistence of STm and *E. coli* O157:H7 in Arabidopsis leaves.

Stomatal defense is an important part of plant innate immunity against plant pathogen attack because it reduces bacterial entry in the leaf apoplast. Stomatal movement is known to require a dynamic guard cell wall, in which synthesis of callose in the guard cells was shown to modulate stomatal movement in the fern *Asplenium nidus* [60]. Thus, guard cell wall reinforcement mediated by EXO70H4 may be a dynamic process that is activated under plant interaction with enterobacteria, as *exo70h4–3* guard cells were impaired in stomatal closure only after inoculation with the human pathogens STm 14,028 s and *E. coli* O157:H7 (Fig. 2). Additionally, EXO70H4 is part of an exocyst complex at the cell plasma membrane, which contains proteins that are conserved among all eukaryotes [61–63], suggesting strengthening of the cell barriers is a common defense mechanism activated by STm and enterohemorrhagic *E. coli* in plants and animals. Therefore, it is possible that guard cells differently respond to human and plant pathogens, in which callose deposition is a plant defense mechanism that may have greater importance in the stomatal defense triggered by human pathogens than by *Pst* DC3118. Further investigation of the molecular components involved in this specific guard cell response to bacteria would contribute to the development of new strategies to diminish fresh produce contamination by STm and *E. coli.*

Interestingly, *E. coli* O157:H7 induces a stronger stomatal defense in the *fls2_SAIL* mutant in comparison to Col-0 (Fig. 4). These corroborate with the notion that other MAMPs (e.g., lipopolysaccharide that is also involved in stomatal immunity [42];), may have a more prominent role in plant perception of *E. coli* O157:H7 than its flagellin. Downstream of MAMP perception however, an intact SA responsive pathway is required for stomatal defense against *E. coli* O157:H7, as well as against STm 14,028 s and *Pst* DC3118 (Fig. 4). This result indicates that SA biosynthesis by ICS1 and signaling through NPR1 are a common mechanism for stomatal defense against phytopathogens [55] and enterobacteria (Fig. 4).

Previous studies have identified a large set of Arabidopsis genes regulated by *E. coli* O157:H7 in a flagellin-dependent manner [43, 45], indicating that this human pathogen activates basal defense mechanisms in the plant apoplast. Likewise, we found that STm SL1344 modulates plant immune responses in Arabidopsis adult leaves (Fig. 1), similar to STm 14,028 s in Arabidopsis seedlings [32, 38]. Among the activated processes, genes involved in ROS burst and cell wall modifications were overrepresented by genes induced after STm SL1344 inoculation, suggesting that these processes play a role in plant basal defense against this human pathogen. A common cell wall modification induced by microbes in plants is the deposition of callose in the infected area reviewed by [49]; a process induced by both SA and flagellin to suppress bacterial growth [64, 65].

Interestingly, flagellin induces the expression of *EXO70H4*, *NPR1* and *ICS1* [66]. Furthermore, EXO70H4 is essential for the secretion of the stress-inducible callose synthase CalS12, also known as POWDERY MILDEW RESISTANT4 (PMR4), which contributes to cell wall strengthening of leaf trichomes [48, 66]. Expression of *PMR4* is strongly induced by SA in a NPR1-dependent manner [58]. These findings indicate that EXO7H4 could function dependently of NPR1. Indeed, we observed that EXO70H4, together with NPR1 and ICS1, were all involved in callose deposition in response to STm 14,028 s, *E. coli* O157:H7, and *Pst* DC3118 (Fig. 3 and Fig. 5). Additionally, decreased callose deposition in the *exo70h4–3* mutant was associated with its compromised apoplastic immunity against STm 14,028 s and *Pst* DC3118 (Fig. 2). We have not observed increased *E. coli* O157:H7 titer in the *exo70h4–3* mutant. However, it is possible that either EXO70H4 has no function in apoplastic immunity against *E. coli* O157:H7 or this bacterium induces redundant apoplastic immune responses in Arabidopsis leaves. Nonetheless, these results support the role of EXO70H4, NPR1, and ICS1 in the control of persistence of human pathogens in leaves at the pre- and/or post-invasion stage of infection (Figs. 2 and 4), and suggest that the plant defense mediated by these proteins involves callose production.

## Conclusion

Stomatal defense against STm and *E. coli* O157:H7 have common mechanisms, requiring EXO70H4, the SA signaling components NPR1 and ICS1, as well as the MAMP receptor FLS2, differently from stomatal defense against *Pst* DC3118 that does not involve EXO70H4. By contrast, STm 14,028 s and *Pst* DC3118 induce similar responses in the leaf mesophyll, as EXO70H4, NPR1, ICS1, and FLS2 are involved in decreasing bacterial populations inside Arabidopsis leaves. Moreover, EXO70H4, NPR1, and ICS1 mediated basal defense via callose deposition is a common plant response against both human and plant pathogens. Although, EXO70H4 is not sufficient to inhibit *E. coli* O157:H7 colonization of the leaf apoplast.

This study provides new insights into genetic mechanism of plant defense against two enterobacteria relevant to public health. Understanding the mechanisms underlying bacterium persistence in edible leaves may facilitate implementation of preventive measures to control foodborne diseases and ultimately improve the quality, safety, and marketability of fresh produce.

## Methods

### Plant material and growth conditions

The seeds of *Arabidopsis thaliana* (L. Heyhn.) wild type ecotype Columbia (Col-0, ABRC stock CS60000) and the 4 derived mutants (Additional file 6; Additional file 8) were sown in a 1:1:1 v:v:v mixture of growing medium (Redi-earth plug and seedling mix, Sun Gro), fine vermiculite, and perlite and grown in controlled environmental chambers at 22 ± 2 °C, 60 ± 10% relative humidity, and a 12-h photoperiod under light intensity of 100 μmol.m^− 2^.s^− 1^. For all experiments, four- to five-week old plants were used.

### Bacterial strains and culturing conditions

*Pseudomonas syringae* pv. *tomato* (*Pst*) strain DC3118 (a coronatine-deficient mutant of the *Pst* DC3000 wild type [67];), *Escherichia coli* serotype O157:H7 strain 86–24 [68] and *Salmonella enterica subsp. enterica* serovar Typhimurium strains SL1344 and 14,028 s [69] were used in this study. Bacterial cultures were grown in Low Salt Luria-Bertani medium (LSLB; 10 g/L tryptone, 5 g/L yeast extract, 5 g/L NaCl, pH = 7.0) at 28 °C for all experiments. Cells were freshly streaked on solid medium from frozen glycerol stocks prior to inoculum preparation. The culture medium was supplemented with streptomycin (50 μg/mL) to grow *E. coli* O157:H7, spectinomycin (100 μg/ml) to grow STm SL1344, or rifampin (100 μg/mL) and kanamycin (50 μg/mL) to grow *Pst* DC3118. STm 14,028 s has no resistance to antibiotics.

### Array hybridization assay

A total of 30 Arabidopsis Col-0 plants were grown as described above. STm SL1344 was cultured in liquid LSLB medium and incubated overnight in an orbital shaker at 30 °C. Inoculum was prepared by harvesting bacterial cells when the culture reached OD_600_ of 0.8–0.9. The inoculum with a final concentration of 1 × 10^8^ CFU.mL^− 1^, supplemented with 0.004% Silwet, was vacuum-infiltrated into the leaf intercellular space of 15 plants as previously described [70]. As mock control, 15 plants were vacuum-infiltrated with water with 0.004% Silwet L-77. After inoculation, plants were kept at 25 °C for 7 h until leaves were collected for RNA extraction using TRizol® reagent (Life Technologies, Grand Island, NY) according to manufacturer’s instruction. Total RNA was quantified through NanoDrop 2000c spectrophotometer (Thermo Scientific, Rockford, IL). RNA quality was assessed by using the Agilent 2010 Bioanalyzer.

Arabidopsis Affymetrix GeneChip ATH1 (Thermo Scientific, Rockford, IL) array hybridizations were conducted in three biological replicates (i.e., independent repetitions of the whole experiment, composed of 5 plants per treatment) at Michigan State University Research Technology Support Facility exactly as described by 43. Raw data of the biological replicates is available in a MIAME-compliant format at the Nottingham Arabidopsis Stock Centre (NASC; http://nasc.cott.ac.uk/) under the Experiment ID NASCARRAYS-674.

### Differential gene expression analysis

Gene expression data were normalized using Robust Multichip Averaging (RMA) normalization across all biological replicates, using the “affylmgui” package (version 1.10.4), available as part of the Bioconductor software package for R [71]. Pairwise Log_2_ fold change values (average of the three biological replicates) were calculated between mock- and STm SL1344-inoculated leaves based on the RMA-normalized relative expression values using Microsoft Excel software (Version 2010). Additionally, a Z-ratio-based approach was used to identify differentially expressed genes according to the methods outlined in 46. The Z-ratio approach determines which genes have significantly higher fold changes than other genes in the dataset. A Z-ratio cutoff of 2.33 (Additional file 1) was used to call approximately 2% of the genes in each dataset as being differentially expressed.

Differentially expressed genes were categorized based in their Gene Ontology (GO) annotations using the Singular Enrichment Anlysis (SEA) tool from agriGO v2.0 (http://systemsbiology.cau.edu.cn/agriGOv2/index.php; [72]). GO enrichment was obtained by using the reference dataset gene model TAIR10 (28,362 genome locus) and the complete GO gene ontology type. Statistical significance was detected with the Fisher’s exact test followed by the Yekutieli-False Discovery Rate multiple test correction (FDR < 0.05).

### Microarray data validation by RT-qPCR

Total RNA was extracted from leaves using RNeasy Plant Mini Kit (Qiagen Inc., Valencia, CA) and quantified using a NanoDrop spectrophotometer (Thermo Scientific, Rockford, IL). Total RNA (1 μg) was synthesized into cDNA using the Takara RNA PCR kit (AMV) (Clontech, Montain View, CA) and diluted to a final concentration of 50 ng.μL^− 1^. The Reverse Transcription Quantitative PCR (RT-qPCR) reaction (20 μL) was performed with 10 μL of iTaq Fast SYBR Green Supermix (BioRad, Hercules, CA), 2 μL of cDNA template from the reverse transcriptase reaction described above, and 200 nM of reverse and forward gene-specific primers. Reactions were carried out in an Applied Biosystems 7300 thermocycler (Applied Biosystems, Foster City, CA) using the following cycling parameter: 1 cycle 95 °C for 5 min and 40 cycles of 95 °C for 10 s and 60 °C for 30 s. A dissociation curve was determined for every reaction to confirm the presence of a single amplicon indicating the lack of primer dimers and non-specific products, and that RNA samples were free of DNA contamination. Gene expression levels relative to the mock-inoculated control were calculated using the ΔΔCt method [73] considering the expression of the housekeeping gene *ACT8* as the internal control and the expression value of the mock-treated samples was set as 1. Two biological replicates (each composed of a bulk of three leaves of one plant) and three technical replicates were performed and statistical significance between the means was calculated with Student’s *t*-test.

Gene-specific primer sets that span an intron region were designed using the primer quest software from IDT-SciTools (http://www.idtdna.com/Primerquest/Home/Index). Efficiencies of each pair of primer were evaluated by calculating the linear regression between the five-fold serial dilution of a cDNA pool and the cycle threshold (CT) values. Only primer sets with a correlation coefficient (R^2^) > 0.97 were used. Gene-specific primer sequences are described in the Additional file 9. Two biological replicates and three technical replicates were performed.

### Genotyping of Arabidopsis mutants

To determine the T-DNA insertion or point mutation for each Arabidopsis mutant (Additional file 8), around 5 mg of fresh leaf tissue of one plant of each genotype was grounded in 200 μL of Edwards Solution [74] for DNA extraction. PCR reaction (25 μL) was performed with 1 U DNA polymerase Gotaq® (Promega, WI, USA), 1x enzyme buffer, 1.5 mM MgCl_2_, 200 μM dNTP, 1 μL of gDNA template, and 400 nM of reverse and forward specific primers (Additional file 8). Reactions were carried out using the following cycling parameter: 1 cycle 95 °C for 5 min and 40 cycles of 95 °C for 30 s, 52 °C for 30 s and 72 °C for 1 min, with final extension of 72 °C for 10 min. Mutants *sid2–2* and *npr1–1* were previously obtained using fast neutrons and ethylmethane sulfonate methods, respectively. Thus, the rearrangement and point mutation in these plants were verified by either presence or absence of the amplicon for *sid2–2* [51] or by an additional step of restriction enzyme digestion with the enzyme *Nla*III (New England Biolabs, Ipswich, MA) after PCR amplification for *npr1–1* [52]. Amplicons were visualized after gel electrophoresis using the UV light on a C300 imaging system (Azure Biosystems, CA, USA).

### Stomatal bioassay

To examine the stomatal immunity of mutant plants against *Pst* DC3118, *E. coli* O157:H7, and STm 14,028 s, stomatal bioassays were conducted as previously described [75]. Briefly, three leaves of four- to five-week old plants were floated in water (mock control) or in 1 × 10^8^ CFU.mL^− 1^ of *Pst* DC3118, *E. coli* O157:H7, or STm 14,028 s. Experiments initiated 3 h after dawn to ensure that stomata were open. Floating leaves were kept at 25 °C and light intensity of 100 μmol.m^− 2^.s^− 1^ for the duration of the experiment. Stomatal images and aperture width measurements were obtained with a Nikon Eclipse 80i fluorescent microscope (Nikon Corporations, Shinagawa-ku, Tokyo, Japan), equipped with long-distance objectives. Data points represent the mean of two independent biological replicates, which were composed of 60 stomata of three leaves of one plant per treatment (*n* = 120) ± standard error (SE). Statistical analyses of each bacterium (or water) treatment, in each mutant in relation to the wild type Col-0 were performed using the Student’s *t-*test.

### Bacterial pathogenesis assays

Bacterial strains were cultured at 28 °C in LSLB medium supplemented with appropriate antibiotics until an OD_600_ of 0.8 to 1.0 was reached. To prepare the inoculum, bacteria were collected by centrifugation and suspended in water to a final concentration of 1 × 10^6^ CFU.mL^− 1^ and supplemented with 0.008% Silwet L-77 (Lehle Seeds Co., Round Rock, TX). Plants were vacuum-infiltrated with the same inoculum to ensure uniform inoculation across the plant genotypes tested. Inoculated plants were immediately incubated under the following conditions: 25 °C, 80 ± 10% relative humidity, 12 h of photoperiod (100 μmol.m^− 2^.sec^− 1^) for the duration of the experiment. Bacterial population in the plant apoplast was determined as previously described [70, 76]. Data points represent the mean (n = 12) ± SE of three leaves (each with two technical replicates) of one plant per data point in two independent experiments. Statistical analyses were performed by comparing the bacterium titer in each mutant with that of the wild type Col-0 using the Student’s *t-*test.

### Callose deposition measurements

Callose staining was performed as described previously [77]. Briefly, fully expanded leaves of four- to five-week-old plants were syringe-infiltrated with 1 × 10^8^ CFU.mL^− 1^ of *Pst* DC3118, STm 14,028 s, *E. coli* O157:H7, or water (mock control). Infiltrated leaves were harvested 7 hpi, cleared with 95% ethanol overnight, rehydrated with 50% ethanol and 150 mM K_2_HPO_4_, and stained with 0.01% Aniline Blue dissolved in 150 mM K_2_HPO_4_. Stained material was mounted in 40% glycerol and examined using a Nikon Eclipse 80i fluorescent microscope (Nikon Corporations, Shinagawa-ku, Tokyo, Japan), equipped with a DAPI filter (358-nm excitation and 461-nm emission) and a digital camera. Callose deposition area (mm^2^/cm^2^ of leaf) was quantified by analyzing the digital images using the Binary Area measurement tool and ROI statistics of the software Nikon NIS-Elements AR version 4.13. Data points are representative of two experiments performed independently with similar results. Each experiment was conducted with three to four biological replicates each (one plant per biological replicate per treatment), and an average of 8 to 10 pictures per biological replicate (*n* = 18 to 37) ± SE. Statistical analysis was performed using the Student’s *t-*test to compare the mean callose area in each mutant plant with the wild type plant Col-0.

### ROS production measurements

ROS detection was monitored by a luminol-based assay [78]. Young, fully expanded leaves of four- to five-week-old plants were used for this assay. A minimum of 8 leaf discs (4 mm in diameter) of three plants per genotype/treatment were cut with a cork borer and individually incubated overnight in 150 μL of sterile water on an opaque white 96-well plates (Thermo Scientific, Rockford, IL). In the next day, the water was replaced with a solution containing 34 μg/mL luminol (Sigma-Aldrich, St. Louis, MO, USA), 20 μg/mL horseradish peroxidase type VI (HRP, Sigma-Aldrich, St. Louis, MO, USA) and 1 × 10^8^ CFU.mL^− 1^ of boiled *Pst* DC3118, STm 14,028 s, or *E. coli* O157:H7, or water as a mock control. Luminescence was recorded over 60 min using a Synergy™ H1 microplate reader (Biotek, Winooski, VT, USA) and analyzed using the plate reader software Biotek Gen5 (Biotek, Winooski, VT, USA). Data points are the mean of three independent biological replicates (*n* = 21 to 37) ± SE.

## Supplementary information

**Additional file 1.** Z-ratio analysis of the microarray dataset. (a) Normalized Z-ratio of array intensity data shows a normal distribution of all genes expressed in the STm SL1344-treated leaves as compared to the mock control. The 2% extremes of the bell-shape curve reveal the genes with significant differential expression. (b) Linear regression between relative gene expression calculated with two methods, Z-ratio and Log_2_ fold change, shows high positive correlation (R^2^ = 0.9676).

**Additional file 2.** Relative gene expression analysis (mock- versus SL1344-treated leaves) using Log2 Fold Change and Z-ratio methods. Gene expression for each gene was calculated based on an average of three biological replicates. A total of 541 array probes corresponding to 585 genes were identified as differentially expessed based on Z-ratio analysis (the extreme 2% of up or down-regulated). Genes hilightes in bold had their expression evaluated by RT-qPCR.

**Additional file 3. **Validation of microarray analysis by RT-qPCR. Arabidopsis leaves were infiltrated with STm SL1344 (1 × 10^8^ CFU.mL^− 1^) or water as a mock control. Expression of randomly selected genes was normalized to the expression of the housekeeping gene *ACT8* (AT1G49240). Gene expression levels in the STm 1344-treated samples relative to the mock-treated samples (value set as 1) were calculated using the ΔΔCt method [73]. Results are shown as average (*n* = 6 ± SE) and statistical difference between the means (STm SL1344 vs. mock) was determined using Student’s *t*-test (* = *p* < 0.05, ** = *p* < 0.01, *** = *p* < 0.001, ns = non-significant)*.*

**Additional file 4.** GO Single Enrichment Analysis (SEA) of genes up-regulated (310 genes) or down-regulated (275 genes) by SL1344, using the online tool AgriGO. Only GO terms that contained more than 5 entries and were significantly over-represented (FDR < 0.05) in the query dataset are shown. Query items indicate the number of SL1344-regulated genes in each GO term, and background items indicate the number of annotated genes in the Arabidopsis genome (TAIR10) for each GO term. Statistical significance was calculated with Fisher’s exact test and Yekutieli-False Discovery Rate (FDR) multiple test correction. The ontology terms used are Biological Process (P), Molecular Function (F), and Cellular Component (C). Terms in bold are present in both up- and down-regulated datasets.

**Additional file 5.** Hierarchical organization of all 11 GO terms enriched in both STm up- and down-regulated gene datasets.

**Additional file 6. **Arabidopsis mutant genotyping. (a) Genomic DNA for each T-DNA insertion mutant plant (*fls2_SAIL* or *exo70h4–3*) and the wild type Col-0 was used as a template in PCR amplification with gene-specific primers listed in the Additional file 8. Reactions loaded onto lane 1 contained the LP (left primer) and RP (right primer) set to amplify the wild type allele in Col-0, whereas reactions loaded onto lane 2 contained a T-DNA specific primer and the RP primer to amplify the mutant allele. (b) The *sid2–2* mutant was created with fast neutron [51] and the point mutation was verified by the absence of amplification using gene-specific primers. Amplification of the *ACT2* gene was used as a positive control for the PCR amplification. (c) The *npr1–1* mutant was created with ethylmethane sulfonate [52] and the point mutation was verified by digesting the PCR amplicon with the restriction enzyme *Nla*III.

**Additional file 7. **Statistical analyses for bacterial mediated-stomatal closure shown in Fig. 2a and Fig. 4a. Statistical analyses were performed by comparing water- with bacterium-treated plants using the Student’s t-test. All comparisons showed statistical significance evidenced by the low *p*-value, except for the fls2-SAIL mutant when comparing water with Pst DC3118 treatment.

**Additional file 8.** List of Arabidopsis mutants and primer sequences used to validate the mutation.

**Additional file 9.** Sequence of primers used to validate the microarray results by RT-qPCR analysis.

## Data Availability

Microarray data is available in the MIAME-compliant format at the Nottingham Arabidopsis Stock Centre. Raw data can be downloaded from http://bar.utoronto.ca/NASCArrays/index.php?ExpID=674 under the Experiment ID NASCARRAYS-674.

## Abbreviations

ACT2: ACTIN 2
cDNA: Complementary DNA
CT: Cycle Threshold
DAI: Day(s) After Inoculation
DNA: Deoxyribonucleic Acid
dNTP: Deoxynucleotide Triphosphate
EXO70H4: EXOCYST SUBUNIT EXO70 FAMILY PROTEIN H4
FDOSS: Foodborne Disease Outbreak Surveillance System
FDR: False Discovery Rate
Flg15^*e. coli*^: 15 amino acid-peptide derived from the *Escherichia coli* flagellin
FLS2: FLAGELLIN SENSING 2
FSMA: Food Safety Modernization Act
gDNA: Genomic DNA
GO: Gene Ontology
hpi: Hours Post Inoculation
ICS1: ISOCHORISMATE SYNTHASE 1
LSLB: Low Salt Luria-Bertani
MAMP: Microbe-Associated Molecular Patterns
MTI: MAMP-Triggered Immunity
NPR1: NON-EXPRESSER OF PATHOGENESIS-RELATED GENE 1
OD: Optical Density
PCR: Polymerase Chain Reaction
Pst: *Pseudomonas syringae* pv. *tomato*
R^2^: Correlation Coefficient
RMA: Robust Multichip Averaging
RNA: Ribonucleic Acid
ROS: Reactive Oxygen Species
RT-qPCR: Reverse Transcriptase-quantitative PCR
SA: Salicylic Acid
SE: Standard Error
SEA: Single Enrichment Analysis
Seflg22: 22 amino acid-peptide derived from the *Salmonella enterica* flagellin
STm: *S. enterica* serovar Typhimurium
TAIR: The Arabidopsis Information Resource
UV: Ultraviolet
