# Supplementary material for: Novel molecular components involved in callose-mediated Arabidopsis defense against Salmonella enterica and Escherichia coli O157:H7
Source: BMC Plant Biol. 2020 Jan 8;20:16. doi: 10.1186/s12870-019-2232-x (PMC6950905; doi:10.1186/s12870-019-2232-x)
Supplement: Supplementary file 1 — Additional file 1. Z-ratio analysis of the microarray dataset. (a) Normalized Z-ratio of array intensity data shows a normal distribution of all genes expressed in the STm SL1344-treated leaves as compared to the mock control. The 2% extremes of the bell-shape curve reveal the genes with significant differential expression. (b) Linear regression between relative gene expression calculated with two methods, Z-ratio and Log2 fold change, shows high positive correlation (R2 = 0.9676). [file 12870_2019_2232_MOESM1_ESM.ppt]

## Slide 1
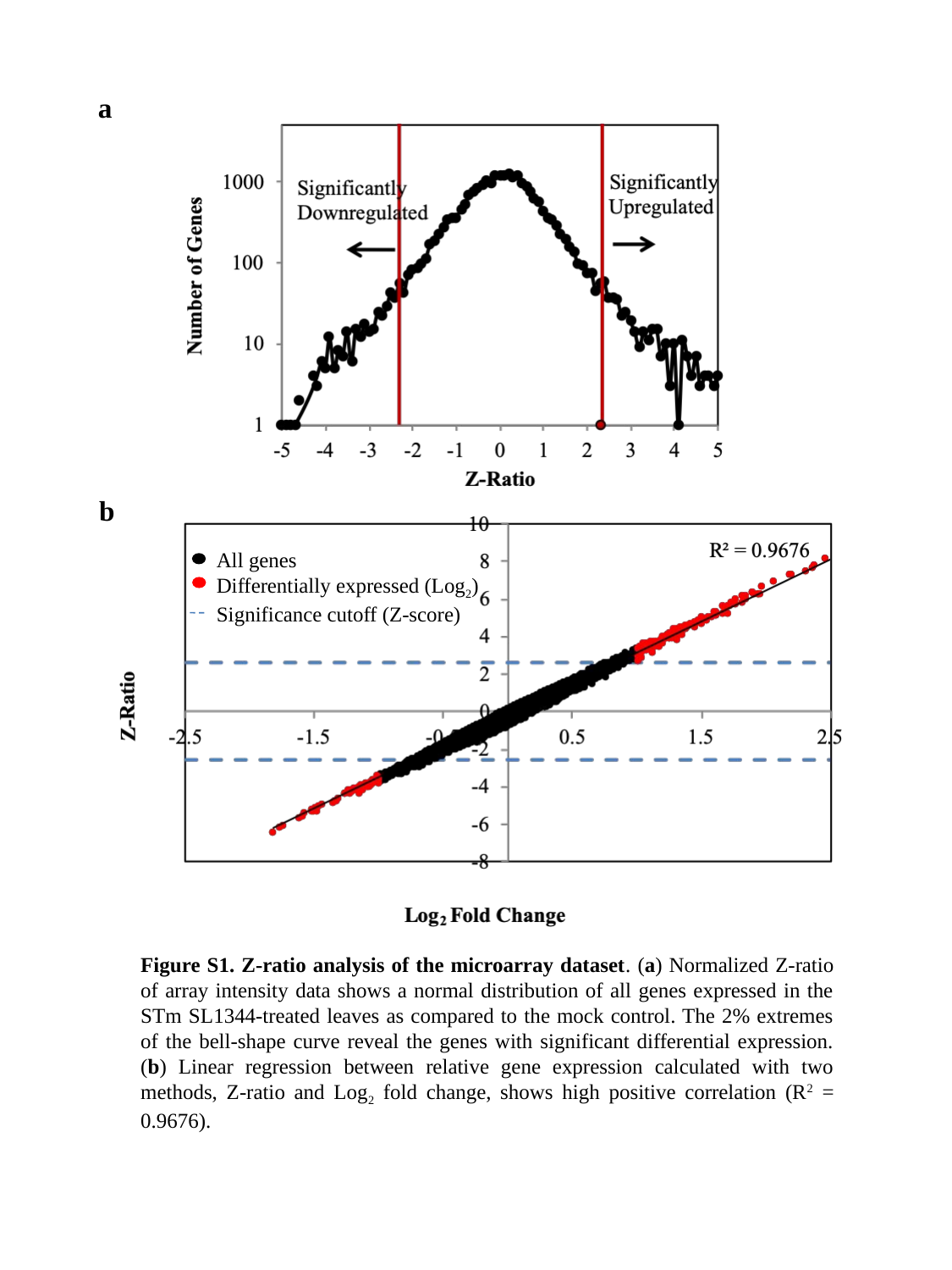

a
b
All genes
Differentially expressed (Log2)
Significance cutoff (Z-score)
Figure S1. Z-ratio analysis of the microarray dataset. (a) Normalized Z-ratio of array intensity data shows a normal distribution of all genes expressed in the STm SL1344-treated leaves as compared to the mock control. The 2% extremes of the bell-shape curve reveal the genes with significant differential expression. (b) Linear regression between relative gene expression calculated with two methods, Z-ratio and Log2 fold change, shows high positive correlation (R2 = 0.9676).
