# Supplementary material for: Novel molecular components involved in callose-mediated Arabidopsis defense against Salmonella enterica and Escherichia coli O157:H7
Source: BMC Plant Biol. 2020 Jan 8;20:16. doi: 10.1186/s12870-019-2232-x (PMC6950905; doi:10.1186/s12870-019-2232-x)
Supplement: Supplementary file 3 — Additional file 3. Validation of microarray analysis by RT-qPCR. Arabidopsis leaves were infiltrated with STm SL1344 (1 × 10^8^ CFU.mL− 1) or water as a mock control. Expression of randomly selected genes was normalized to the expression of the housekeeping gene ACT8 (AT1G49240). Gene expression levels in the STm 1344-treated samples relative to the mock-treated samples (value set as 1) were calculated using the ΔΔCt method [73]. Results are shown as average (n = 6 ± SE) and statistical difference between the means (STm SL1344 vs. mock) was determined using Student’s t-test (* = p < 0.05, ** = p < 0.01, *** = p < 0.001, ns = non-significant). [file 12870_2019_2232_MOESM3_ESM.pptx]

## Slide 1
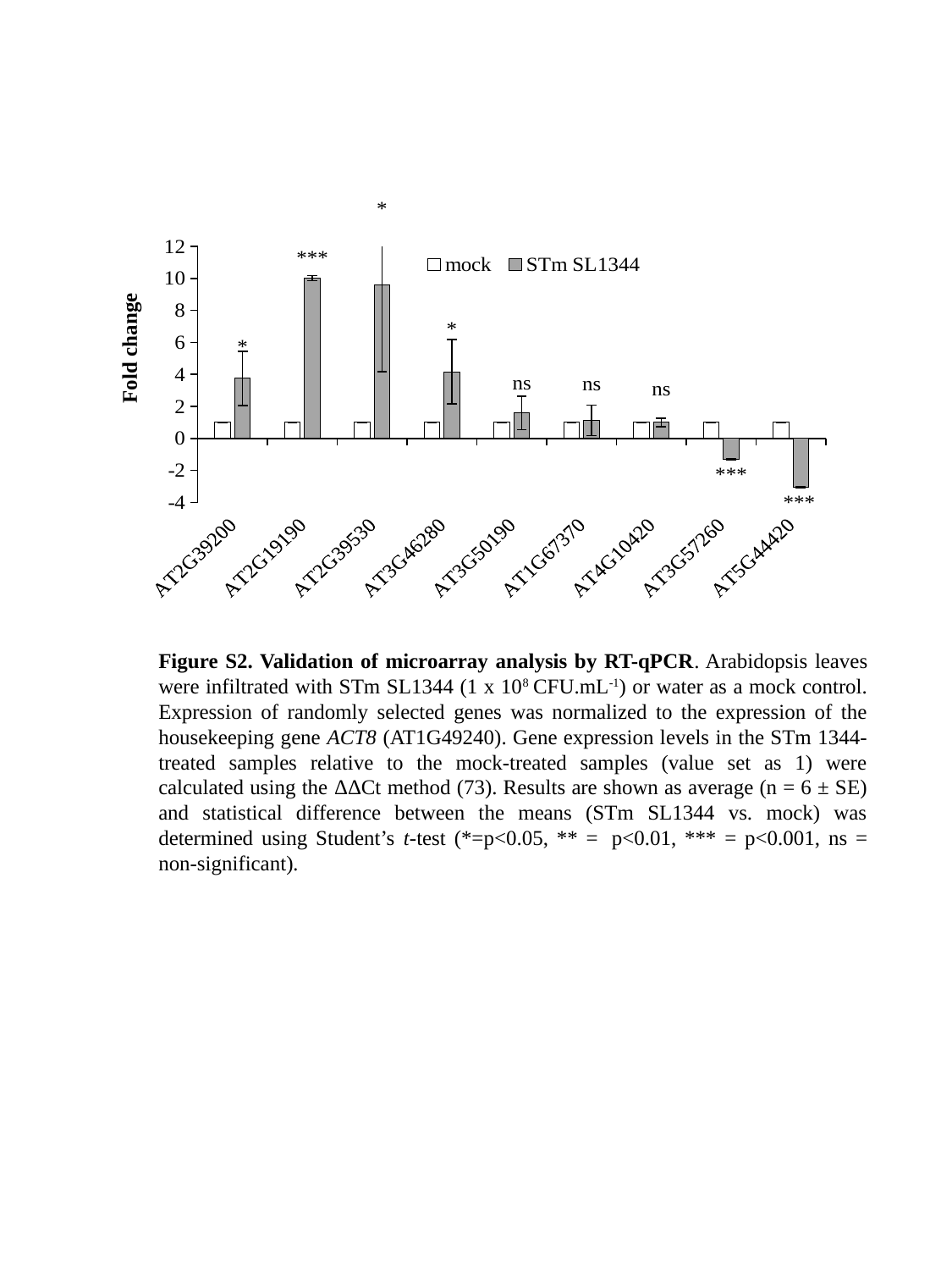

### Chart
| Category | mock | STm SL1344 |
|---|---|---|
| AT2G39200 | 1.0 | 3.753742313359355 |
| AT2G19190 | 1.0 | 10.01060273919264 |
| AT2G39530 | 1.0 | 9.600039294196398 |
| AT3G46280 | 1.0 | 4.167685623557274 |
| AT3G50190 | 1.0 | 1.5892281567727113 |
| AT1G67370 | 1.0 | 1.132010205034072 |
| AT4G10420 | 1.0 | 1.000474294759466 |
| AT3G57260 | 1.0 | -1.3188285107593747 |
| AT5G44420 | 1.0 | -3.0762609140044495 |Fold change
Figure S2. Validation of microarray analysis by RT-qPCR. Arabidopsis leaves were infiltrated with STm SL1344 (1 x 10^8^ CFU.mL-1) or water as a mock control. Expression of randomly selected genes was normalized to the expression of the housekeeping gene ACT8 (AT1G49240). Gene expression levels in the STm 1344-treated samples relative to the mock-treated samples (value set as 1) were calculated using the ΔΔCt method (73). Results are shown as average (n = 6 ± SE) and statistical difference between the means (STm SL1344 vs. mock) was determined using Student’s t-test (*=p<0.05, ** =  p<0.01, *** = p<0.001, ns = non-significant).
