# Supplementary material for: Novel molecular components involved in callose-mediated Arabidopsis defense against Salmonella enterica and Escherichia coli O157:H7
Source: BMC Plant Biol. 2020 Jan 8;20:16. doi: 10.1186/s12870-019-2232-x (PMC6950905; doi:10.1186/s12870-019-2232-x)
Supplement: Supplementary file 5 — Additional file 5. Hierarchical organization of all 11 GO terms enriched in both STm up- and down-regulated gene datasets. [file 12870_2019_2232_MOESM5_ESM.pptx]

## Slide 1
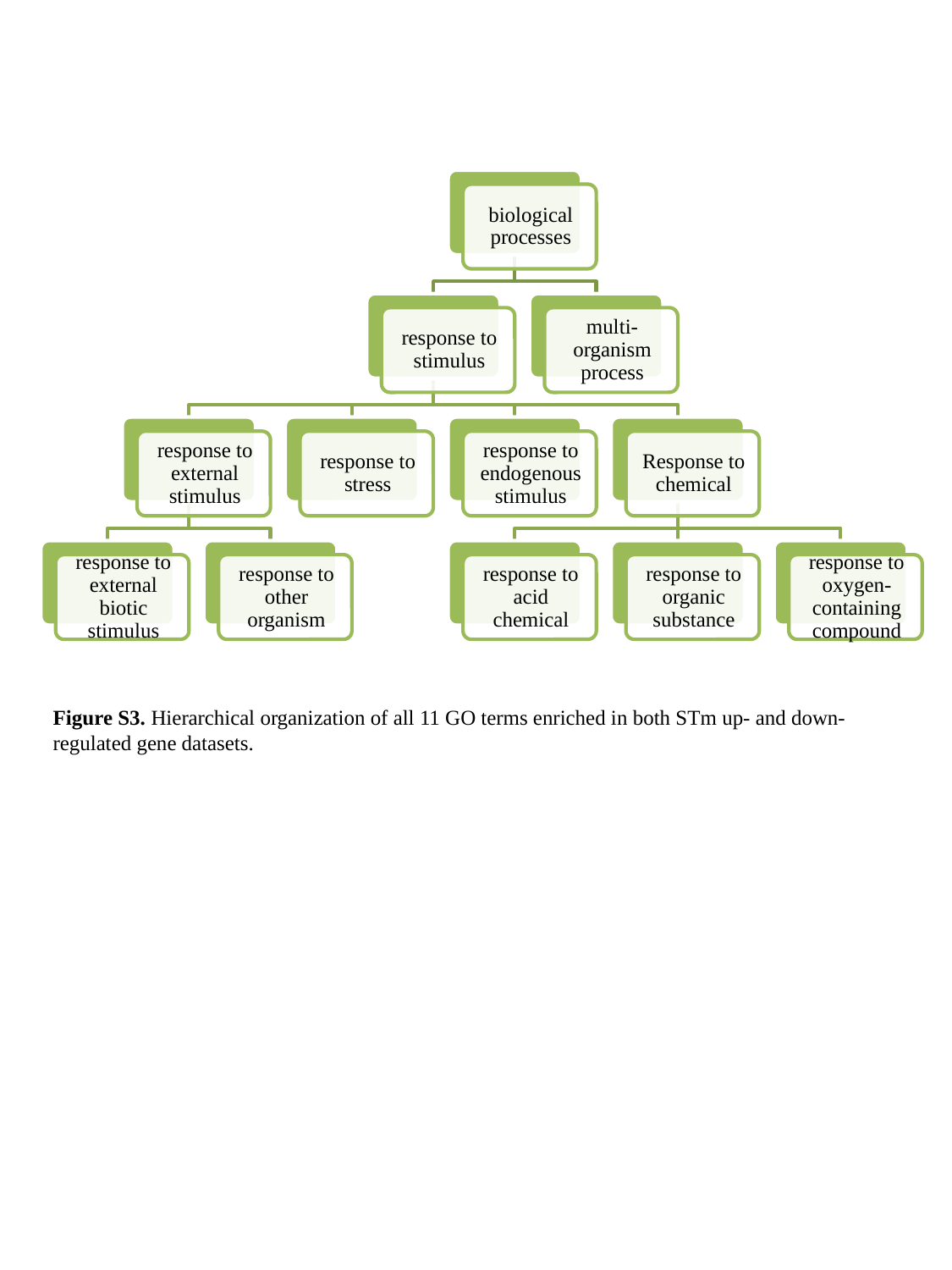

Figure S3. Hierarchical organization of all 11 GO terms enriched in both STm up- and down-regulated gene datasets.
