# Supplementary material for: Novel molecular components involved in callose-mediated Arabidopsis defense against Salmonella enterica and Escherichia coli O157:H7
Source: BMC Plant Biol. 2020 Jan 8;20:16. doi: 10.1186/s12870-019-2232-x (PMC6950905; doi:10.1186/s12870-019-2232-x)
Supplement: Supplementary file 6 — Additional file 6. Arabidopsis mutant genotyping. (a) Genomic DNA for each T-DNA insertion mutant plant (fls2_SAIL or exo70h4–3) and the wild type Col-0 was used as a template in PCR amplification with gene-specific primers listed in the Additional file 8. Reactions loaded onto lane 1 contained the LP (left primer) and RP (right primer) set to amplify the wild type allele in Col-0, whereas reactions loaded onto lane 2 contained a T-DNA specific primer and the RP primer to amplify the mutant allele. (b) The sid2–2 mutant was created with fast neutron [51] and the point mutation was verified by the absence of amplification using gene-specific primers. Amplification of the ACT2 gene was used as a positive control for the PCR amplification. (c) The npr1–1 mutant was created with ethylmethane sulfonate [52] and the point mutation was verified by digesting the PCR amplicon with the restriction enzyme NlaIII. [file 12870_2019_2232_MOESM6_ESM.pptx]

## Slide 1
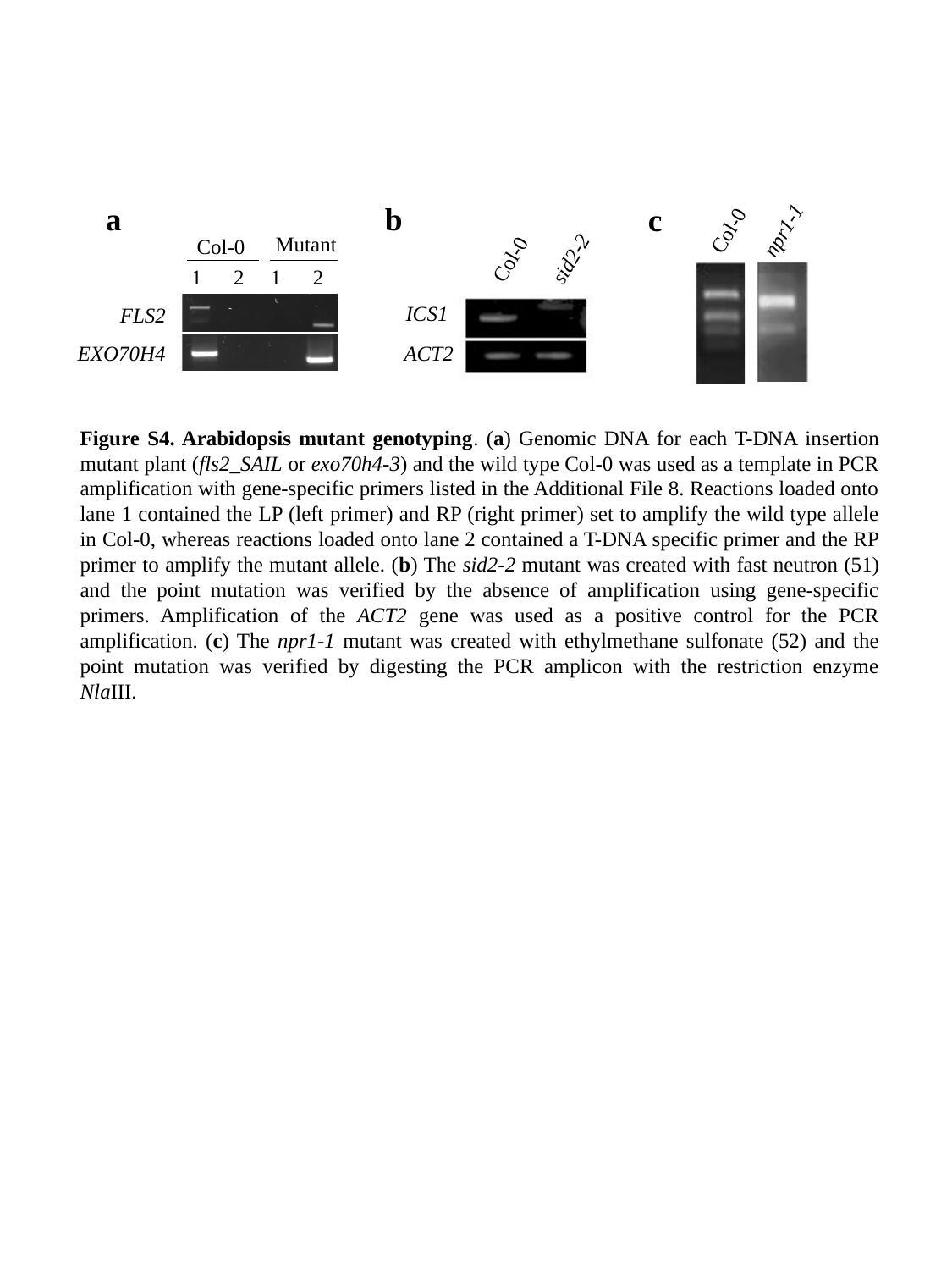

Col-0
npr1-1
a
b
c
Col-0
sid2-2
ICS1
ACT2
Mutant
Col-0
1 2 1 2
FLS2
exo70h4
Figure S4. Arabidopsis mutant genotyping. (a) Genomic DNA for each T-DNA insertion mutant plant (fls2_SAIL or exo70h4-3) and the wild type Col-0 was used as a template in PCR amplification with gene-specific primers listed in the Additional File 8. Reactions loaded onto lane 1 contained the LP (left primer) and RP (right primer) set to amplify the wild type allele in Col-0, whereas reactions loaded onto lane 2 contained a T-DNA specific primer and the RP primer to amplify the mutant allele. (b) The sid2-2 mutant was created with fast neutron (51) and the point mutation was verified by the absence of amplification using gene-specific primers. Amplification of the ACT2 gene was used as a positive control for the PCR amplification. (c) The npr1-1 mutant was created with ethylmethane sulfonate (52) and the point mutation was verified by digesting the PCR amplicon with the restriction enzyme NlaIII.
