# Supplementary material for: Novel molecular components involved in callose-mediated Arabidopsis defense against Salmonella enterica and Escherichia coli O157:H7
Source: BMC Plant Biol. 2020 Jan 8;20:16. doi: 10.1186/s12870-019-2232-x (PMC6950905; doi:10.1186/s12870-019-2232-x)
Supplement: Supplementary file 7 — Additional file 7. Statistical analyses for bacterial mediated-stomatal closure shown in Fig. 2a and Fig. 4a. Statistical analyses were performed by comparing water- with bacterium-treated plants using the Student’s t-test. All comparisons showed statistical significance evidenced by the low p-value, except for the fls2-SAIL mutant when comparing water with Pst DC3118 treatment. [file 12870_2019_2232_MOESM7_ESM.docx]

**Table S3.** Statistical analyses for bacterial mediated-stomatal closure shown in Figures 2a and 4a. Statistical analyses were performed by comparing water- with bacterium-treated plants using the Student’s *t-*test. All comparisons showed statistical significance evidenced by the low p-value, except for the *fls2-SAIL* mutant when comparing water with *Pst* DC3118 treatment.

|  |  | p-values (water versus bacterium treatment) | | |
| --- | --- | --- | --- | --- |
|  |  | *Pst* DC3118 | *E. coli* O157:H7 | STm 14028s |
| Figure 2a | Col-0 | 6.15 x 10^-48^ | 5.17 x 10^-31^ | 7.38 x 10^-40^ |
|  | *exo70h4-3* | 2.30 x 10^-46^ | 5.65 x 10^-07^ | 2.03 x 10^-14^ |
| Figure 4a | Col-0 | 2.65 x 10^-27^ | 6.21 x 10^-43^ | 6.34 x 10^-42^ |
|  | *sid2-2* | 1.53 x 10^-27^ | 7.20 x 10^-15^ | 4.88 x 10^-42^ |
|  | Col-0 | 3.63 x 10^-53^ | 4.52 x 10^-48^ | 5.95 x 10^-47^ |
|  | *npr1-1* | 6.84 x 10^-51^ | 2.18 x 10^-09^ | 1.47 x 10^-11^ |
|  | Col-0 | 6.85 x 10^-36^ | 1.65 x 10^-14^ | 1.38 x 10^-31^ |
|  | *fls2-SAIL* | 6.50 x 10^-01^ | 6.68 x 10^-51^ | 3.08 x 10^-13^ |
